# Structural characterization of alkali-soluble polysaccharides from *Panax ginseng* C. A. Meyer

**DOI:** 10.1098/rsos.171644

**Published:** 2018-03-14

**Authors:** Li Ji, Zhenjing Jie, Xin Ying, Qi Yue, Yifa Zhou, Lin Sun

**Affiliations:** Jilin Province Key Laboratory on Chemistry and Biology of Natural Drugs in Changbai Mountain, School of Life Sciences, Northeast Normal University, Changchun 130024, People's Republic of China

**Keywords:** ginseng, polysaccharides, pectin, hemicellulose, structural analysis

## Abstract

*Panax ginseng* C. A. Meyer (ginseng) has been widely used as a herb and functional food in the world. Polysaccharides are the main active components of ginseng. In this paper, the polysaccharides were sequentially extracted by 50 mM Na_2_CO_3_, 1 M KOH and 4 M KOH from ginseng roots treated sequentially with hot water, α-amylase and ethylenediaminetetraacetic acid extraction. Na_2_CO_3_-soluble ginseng polysaccharide (NGP) was fractionated into one neutral and three acidic fractions by anion exchange and gel permeation chromatography. Fourier transform infrared, NMR and methylation analysis indicated acidic fractions in NGP were highly branched rhamnogalacturonan-I domains, with  → 4)-α-Gal*p*A-(1 → 2)-α-Rha*p*-(1 → disaccharide repeating units as backbone and β-1,4-galactan, α-1,5/1,3,5-arabinan and type II arabinogalactan as side chains. 1-KGP (1 M KOH-soluble ginseng polysaccharide) and 4-KGP (4 M KOH-soluble ginseng polysaccharide) were mainly composed of hemicellulose besides starch-like polysaccharides and minor pectin. Antibody detection, enzymic hydrolysis, high performance anion exchange chromatography and methylation analysis demonstrated xylan was the major component in 1-KGP, while xyloglucan was predominant in 4-KGP. Comparing the polysaccharides obtained by different solvent extractions, we have a comprehensive understanding about total ginseng polysaccharides.

## 1. Introduction

*Panax ginseng* C. A. Meyer (ginseng) is a well-known herb and has been widely used as functional food in the world. Polysaccharides are the major active components of ginseng [1]. Many studies have been conducted on ginseng polysaccharides concerning their purification, structural analysis and bioactivities since 1966. Among these reports, most of the polysaccharides were extracted from ginseng with hot water [2–6]. In recent years, our laboratory has carried out a series of studies about structure--activity relationships of water-soluble ginseng polysaccharides. Our results indicated water-soluble ginseng polysaccharides contained starch-like glucans, homogalacturonan (HG), rhamnogalacturonan-I (RG-I) and arabinogalactan (AG)-type pectin [7]. These polysaccharides exhibited various activities including inhibitory effects against galectin-3 [8], immunomodulatory effects [9], inhibition of cell migration [10] and anti-fatigue activity [11]. Owing to the interference of starch granules, some of the polysaccharides could not be extracted from ginseng with hot water. We then performed an extraction by α-amylase solution after hot water extraction, and polysaccharides with different chemical structures compared with water-soluble ginseng polysaccharides were obtained [12]. As some pectic polysaccharides usually bind Ca^2+^ ions within the lamella of cell walls, forming so-called egg-box structures, they could not be extracted easily with water alone. However, these polysaccharides can be solubilized by using chelating agents. Therefore, we used EDTA solution to further extract these polysaccharides from ginseng roots [13].

Although ginseng has already been extracted by different methods, there might be some non-cellulosic polysaccharides still present in ginseng roots. To get the content and structural information of these non-cellulosic polysaccharides, other extraction methods should be applied. Alkali solutions have been used for obtaining pectin and hemicellulose which are highly branched and cross-linked in cell walls [14]. Therefore, in this study, 50 mM Na_2_CO_3_, 1 M KOH and 4 M KOH solutions were used in sequence to extract polysaccharides from ginseng roots after hot water, α-amylase and EDTA solution treatment. The structures of these alkali-soluble polysaccharides were analysed by NMR, monoclonal antibody and high performance anion exchange chromatography (HPAEC). Based on the results of present and previous studies, we could have a comprehensive understanding of the total polysaccharides in ginseng.

## 2. Material and methods

### 2.1. Materials

Ginseng was cultivated and collected from Changbai Mountain, Jilin Province, China. Sepharose CL-6B was purchased from GE Healthcare (Pittsburgh, USA). DEAE-cellulose was purchased from Shanghai Chemical Reagent Research Institute (Shanghai, China). α-Amylase (E.C.3.2.1.1) from *Bacillus* spp. was purchased from Sigma–Aldrich (St Louis, MO, USA). Endo-1,4-β-glucanase (EC 3.2.1.4) from *Trichoderma longibrachiatum* was purchased from Megazyme (Bray, Ireland). All other reagents were of analytical or high performance liquid chromatography (HPLC) grade.

### 2.2. General methods

Total carbohydrate content was determined by the phenol--sulfuric acid method, using a mixture of major monosaccharides as the standard [15]. Uronic acid content was determined by the *m*-hydroxydiphenyl method, using galacturonic acid as the standard [16]. Starch was detected by the I_2_--KI assay. Monosaccharide composition analysis was performed by HPLC as previously described [7]. Weight-average molecular weights (Mw) were determined by using high performance gel-permeation chromatography (HPGPC) with a TSK-gel G-3000 PWXL column (7.8 × 300** **mm, TOSOH, Japan) coupled to a Shimadzu HPLC system [7]. HPAEC was performed with a Carbo-Pac PA 200 column (3** **mm × 250** **mm, Dionex, Sunnyvale, USA) and an ED electrochemical detector (Dionex, Sunnyvale, USA) working in an amperometric mode [17].

### 2.3. Extraction of polysaccharides from ginseng roots by alkali

Ginseng roots (500** **g) were sequentially extracted with hot water, α-amylase and EDTA solution as described previously, yielding polysaccharide fractions named WGP (water-soluble ginseng polysaccharides), WGPE (water-soluble ginseng polysaccharide extracted by enzyme) and EGP (EDTA-soluble ginseng polysaccharide), respectively [7,12,13]. After that, ginseng roots were further extracted with 4 l of 50** **mM Na_2_CO_3_ solution (containing 20** **mM NaBH_4_) at 4°C for 16** **h. This procedure was performed in duplicate. After filtration, the residues were washed twice with distilled water. The filtrates and washings were combined and centrifuged at 4500** **rpm for 10** **min. The supernatant was neutralized with 50% acetic acid, dialysed against distilled water, and then freeze-dried, yielding Na_2_CO_3_-soluble ginseng polysaccharide (NGP). Ginseng roots were then extracted with 3 l of 1 M KOH solution (containing 20** **mM NaBH_4_) and then 4 M KOH solution (containing 20** **mM NaBH_4_), both at room temperature for 16** **h. The extracts were neutralized with 50% acetic acid, dialysed against distilled water, and then freeze-dried, yielding 1 M KOH-soluble ginseng polysaccharide (1-KGP) and 4 M KOH-soluble ginseng polysaccharide (4-KGP).

### 2.4. Enzymatic hydrolysis

1-KGP or 4-KGP (50** **mg ml^−1^ in distilled water) was hydrolysed by α-amylase (400 U) at 50°C for 24** **h. Then the enzyme was inactivated by heating the reaction mixture at 100°C for 10** **min and removed by centrifugation. The supernatant was dialysed (molecular weight cut-off of 3500** **Da) against distilled water. Polymeric fraction inside of the dialysis tube (named 1-KGP-P and 4-KGP-P) and oligosaccharide fraction outside of the dialysis tube (named 1-KGP-O and 4-KGP-O) were both collected.

1-KGP-P or 4-KGP-P (5** **mg) was suspended in distilled water (1 ml) containing 10 U of endo-1,4-β-glucanase at 40**°**C under slow agitation during 16** **h. After enzyme inactivation by boiling for 10** **min and centrifugation at 10 000** **rpm for 5** **min, solutions were passed through a 0.45 µm filter prior to further HPAEC analysis.

### 2.5. Anion exchange chromatography

NGP (3** **g) was dissolved in distilled water (80 ml), centrifuged and the supernatant was loaded on a DEAE-cellulose column (4.0 × 20** **cm, Cl**^−^**) pre-equilibrated with distilled water. The column was eluted by a stepwise gradient of NaCl aqueous solutions (0.0, 0.1 and 0.4 M) at a flow rate of 5 ml min^−1^. The eluate was collected at 10 ml per tube and assayed for the distribution of total sugars and uronic acids. Fractions of interest were pooled, concentrated, dialysed and freeze-dried to give NGP-N, NGP-1 and NGP-2.

### 2.6. Size exclusion chromatography

Samples (NGP-1, NGP-2, 1-KGP-P or 4-KGP-P) were fractionated on a Sepharose CL-6B (1.5 × 90** **cm) column. Each fraction was dissolved in 0.15 M NaCl, loaded onto the column and eluted with the same buffer at a flow rate of 0.15 ml min^−1^. Fractions (3 ml per tube) were collected and assayed for total sugar and uronic acid contents. The appropriate fractions were combined, concentrated, dialysed and freeze-dried.

### 2.7. Enzyme-linked immunosorbent assay analysis

1-KGP-P or 4-KGP-P (50 µg ml^−1^) in carbonate--bicarbonate buffer (pH 9.6) was coated on 96-well Nunc-Immuno MaxiSorp microtiter plates overnight at 4°C. The coating solutions were removed and 200 µl well^−1^ of 4% (w/v) milk protein in phosphate-buffered saline (MP/PBS) was added to block the plates for 2** **h at room temperature to prevent non-specific binding. The plates were washed and then 100 µl well^−1^ of the primary antibody (LM10, LM 15 and LM 21) was added at a 1 : 50 dilution in MP/PBS. Following incubation for 1.5** **h, the plates were washed and the wells were incubated with anti-rat IgG coupled to horseradish peroxidase (HRP) for an additional 1.5** **h. After washing with PBS, 100 µl well^−1^ of freshly prepared HRP-substrate (18 ml of deionized water, 2 ml of 1 M sodium acetate buffer, pH 6.0, 200 µl of tetramethylbenzidine and 20 µl of 6% (v/v) hydrogen peroxide) was added. The reaction was stopped after 5** **min by the addition of 100 µl well^−1^ of 2 M H_2_SO_4_. Antibody binding was determined by measuring the absorbance at 450** **nm in a micro-plate reader (Bio-Rad, USA) [18].

### 2.8. Fourier transform infrared spectroscopy

Polysaccharides were ground with KBr powder and then pressed into a 1** **mm pellet for Fourier transform infrared (FT-IR) measurements. FT-IR spectra were obtained using a Spectrum Two FT-IR spectrometer in the range of 4000–400 cm**^−^**^1^ (Perkin Elmer, USA).

### 2.9. NMR spectroscopy

Samples (20** **mg) were dissolved in D_2_O (1 ml, 99.8%) and stirred overnight at room temperature. ^13^C NMR spectra were obtained using a Bruker AV600 NMR spectrometer (Bruker Inc., Rheinstetten, Germany) operating at 150** **MHz. Spectra were recorded at 25°C with 57 000 transients. Acetone was used as an internal standard [7].

### 2.10. Methylation analysis

Methylation analysis was carried out according to the method of Needs and Selvendran with a small modification [19]. In brief, polysaccharide sample (5** **mg) was dissolved in dimethylsulfoxide (DMSO; 0.5 ml) and methylated by treatment with a suspension of NaOH/DMSO (0.5 ml) and iodomethane (1.0 ml). The reaction mixture was extracted with CHCl_2_, and then the solvent was removed by vacuum evaporation. Complete methylation was confirmed by the disappearance of the –OH band (3200–3400 cm^−1^) in the FT-IR spectrum. The per-O-methylated polysaccharide was hydrolysed subsequently by using HCOOH (85%, 1 ml) for 4** **h at 100°C and then CF_3_COOH (2 M, 1 ml) for 6** **h at 100°C. The partially methylated sugars in the hydrolysate were reduced by using NaBH_4_ and acetylated. The resulting alditol acetates were analysed by gas chromatography–mass spectrometry (GC-MS; 7890B-5977B, Agilent, USA).

## 3. Results and discussion

### 3.1. Extraction of ginseng polysaccharide by alkali

In our previous reports, ginseng roots were sequentially extracted by using hot water, α-amylase and EDTA solution, yielding polysaccharide fractions named WGP (yield 10.7%), WGPE (yield 9.0%) and EGP (yield 6.0%), respectively [7,12,13]. In this study, ginseng roots which had been extracted by the above-mentioned three solvents were further extracted in succession by using 50** **mM Na_2_CO_3_ solution, 1 M KOH and 4 M KOH solution, respectively, producing polysaccharide fractions named NGP (yield 1.8%), 1-KGP (yield 2.2%) and 4-KGP (yield 2.8%). Among these polysaccharides, 50** **mM Na_2_CO_3_ solution extracted polysaccharide NGP had the lowest yield, while hot water-extracted polysaccharide WGP had the highest yield. The yields for the alkali-extracted polysaccharides were all lower than those extracted with hot water, α-amylase or EDTA solution. I_2_--KI assay showed that except for NGP, all other fractions contained starch-like polysaccharides. NGP contained GalA (27.1%), Ara (28.0%) and Gal (24.9%) as main sugars, with less Glc (13.8%) and Rha (6.2%) (table 1), suggesting it was mainly composed of pectic polysaccharides. These pectins might be covalently cross-linked to the cell wall and could be extracted by Na_2_CO_3_ which broke interpolymeric ester linkages [14]. Monosaccharide compositions of 1-KGP and 4-KGP were very similar. They contained Glc in majority (72.0% for 1KGP and 70.7% for 4KGP). Xyl and Man were present in these fractions, suggesting they might contain hemicellulose such as xyloglucan, xylan or glucomannan [17]. Besides, minor GalA, Gal and Ara were detected in 1-KGP and 4-KGP, indicating small amounts of pectic polysaccharides were also extracted under alkali condition. These pectins might be highly branched and cross-linked to hemicellulose in the cell wall; therefore, they were extracted together by high concentration alkali. The remaining insoluble ginseng root residues were designated as cellulose fraction (approx. 35%).

**Table 1.** Monosaccharide compositions of ginseng polysaccharides extracted by different solvents.

	NGP	1-KGP	4-KGP	WGP	WGPE	EGP
yield (w/w%)	1.8	2.2	2.8	10.7	9.0	6.0
sugar composition (w/w%)						
Glc	13.8	72.0	70.7	78.0	83.4	50.4
GalA	27.1	2.0	1.7	9.4	6.3	24.2
Gal	24.9	4.3	7.5	6.8	4.5	10.5
Ara	28.0	3.8	5.0	3.8	4.8	11.6
Xyl	—	9.1	12.0	—	—	—
Rha	6.2	—	—	1.1	1.0	3.2
Man	—	6.9	2.0	1.0	—	—
total sugar (w/w%)	70.3	68.5	68.0	77.1	72.5	72.1
protein (w/w%)	4.5	3.4	2.3	1.0	0.7	1.1
starch	−	+	+	+	+	+

### 3.2. Purification and structural analysis of NGP

#### 3.2.1. Purification of NGP by anion-exchange and size-exclusion chromatographies

**Figure 1.**
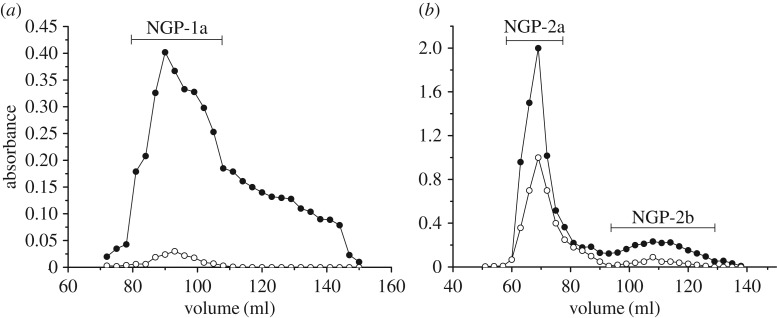
Elution profiles of (*a*) NGP-1 and (*b*) NGP-2 on Sepharose CL-6B column (filled circles, total sugar; open circles, uronic acid).

NGP was first fractionated on a DEAE-cellulose column (Cl**^−^**), eluted stepwise with water, 0.1 and 0.4 M NaCl. One neutral polysaccharide (NGP-N, yield 15.8%) and two acidic polysaccharides (NGP-1, 21.8% and NGP-2, 13.5%) were obtained. NGP-1 (figure 1*a*) and NGP-2 (figure 1*b*) were further fractionated on Sepharose CL-6B column. One purified fraction NGP-1a was obtained from NGP-1, and two fractions NGP-2a and NGP-2b were obtained from NGP-2. NGP-N contained 52.3% Glc, 25.0% Gal and 19.6% Ara, with trace Rha (table 2). As it is not homogeneous, NGP-N was not further studied. NGP-1a, NGP-2a and NGP-2b all contained Gal, Ara, GalA and Rha as main sugars (table 2). The ratios of Rha/GalA in these fractions were 0.68, 0.69 and 0.82, typical for RG-I-type pectin. In NGP-1a and NGP-2b, the content of Gal was double that of Ara, while in NGP-2a, the content of Gal was half that of Ara. High contents of Ara and Gal in these fractions reflected that they contained large number of neutral side chains.

**Table 2.** Molecular weights and monosaccharide compositions of NGP fractions.

			monosaccharide composition (w/w %)				
fraction	total sugar (w/w %)	Mw (kDa)	GalA	Rha	Gal	Ara	Glc
NGP-N	90.5	—	—	2.8	25.0	19.6	52.3
NGP-1a	91.3	123	6.3	4.3	58.8	30.6	—
NGP-2a	89.6	153	19.1	13.2	22.5	45.2	—
NGP-2b	90.0	4.9	8.7	7.1	60.3	23.9	—

#### 3.2.2. Fourier transform infrared analysis of purified fractions from NGP

**Figure 2.**
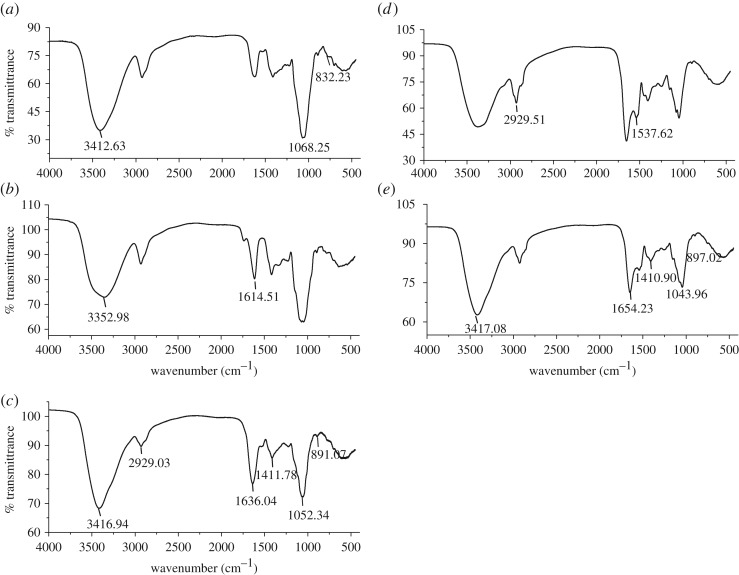
The FT-IR spectra of (*a*) NGP-1a, (*b*) NGP-2a, (*c*) NGP-2b, (*d*) 1-KGP-P and (*e*) 4-KGP-P.

The FT-IR spectra of purified fractions from NGP showed typical carbohydrate characters (figure 2*a–c*). The strong broad band around 3412 cm^−1^ was attributed to the O–H stretching vibration due to intermolecular and intramolecular hydrogen bonds. The absorption at 2929 cm^−1^ was attributed to C–H stretching vibration. The band at 1614 cm**^−^**^1^ coupled with another weaker band at 1411 cm**^−^**^1^ was from the asymmetric and symmetric stretching vibration of the carbonyl groups, respectively. The bands in the region from 1000 to 1200 cm^−1^ were assigned to the absorption of skeletal C–O and C–C vibrations of glycosidic bonds and pyranoid ring. The weak bands at 891 cm**^−^**^1^ and 832 cm**^−^**^1^ indicated the presence of β-linked and α-linked sugar residues [20].

#### 3.2.3. NMR analysis of purified fractions from NGP

**Figure 3.**
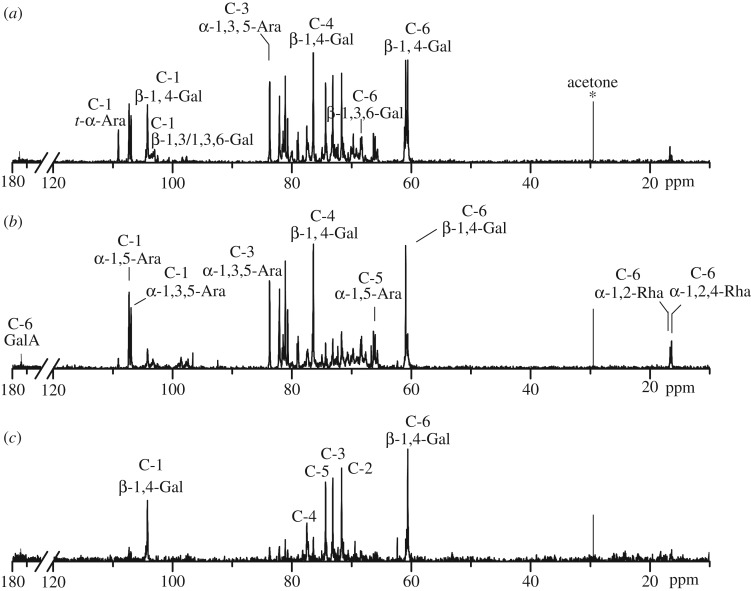
The ^13^C NMR spectra of (*a*) NGP-1a, (*b*) NGP-2a and (*c*) NGP-2b.

The structures of NGP-1a, NGP-2a and NGP-2b were further analysed by ^13^C NMR spectroscopy (figure 3), and the chemical shift assignments are listed in table 3. As can be seen, GalA and Rha showed low intensity signals in these fractions, which were in accordance with their monosaccharide compositions. The anomeric signals at 97.41–98.10** **ppm were assigned to C-1 of α-1,4-GalA, and signals at 173.84–174.53** **ppm were assigned to C-6 of α-1,4-GalA. The two signals at 16.66 and 16.39** **ppm were attributed to C-6 of α-1,2-Rha and α-1,2,4-Rha, respectively [21]. The presence of these resonances confirmed the existence of RG-I-type pectin in NGP-1a, NGP-2a and NGP-2b. β-1,4-Linked Gal residues exhibited six signals at 104.22, 71.68, 73.16, 76.40, 74.36 and 60.06** **ppm, corresponding to their C-1 to C-6 carbons [22]. Low intensity signals at 103.31 and 80.05** **ppm were attributed to C-1 and C-3 of β-1,3/1,3,6-Gal, respectively. The anomeric signals at 107.28 and 106.95** **ppm were assigned to C-1 of α-1,5-Ara and α-1,3,5-Ara, respectively, and the signals at 83.75** **ppm and 79.09** **ppm were assigned to the C-3 carbons of α-1,3,5-Ara and α-1,5-Ara, respectively. Low intensity signal at 109.08** **ppm was attributed to the C-1 of t-α-Ara*f* [23]. NMR results indicated that RG-I domains in NGP-1a, NGP-2a and NGP-2b might be highly branched with β-1,4-galactan, α-1,5/1,3,5-arabinan and type II arabinogalactan side chains. Previous studies also showed that 50** **mM Na_2_CO_3_ mainly solubilized ramified hairy region of pectic polysaccharides from squash cell walls [24] and *Psidium cattleianum* [14].

**Table 3.** ^13^C NMR spectral assignments of NGP-1a, NGP-2a and NGP-2b.

		chemical shifts, *δ* (ppm)					
fraction	sugar residues	C-1	C-2	C-3	C-4	C-5	C-6
NGP-1a	→4)-α-Gal*p*A-(1→	97.95	66.34	68.31	77.52	68.49	174.12
	→4)-β-Gal*p*-(1→	104.22	71.68	73.16	76.42	74.36	60.60
	→3)-β-Gal*p*-(1→	103.31	—	80.05	—	—	60.60
	→3,6)-β-Gal*p*-(1→	103.31	—	80.05	—	—	68.49
	→5)-α-Ara*f*-(1→	107.28	80.22	79.09	82.08	66.06	
	→3,5)-α-Ara*f*-(1→	106.95	79.82	83.75	80.72	66.34	
	t-α-Ara*f*-(1→	109.08	81.18	78.97	80.72	60.94	
	→2/2,4)-α-Rha*p*-(1→	98.15	—	—	—	—	16.66/16.39
NGP-2a	→4)-α-Gal*p*A-(1→	97.41	66.34	68.31	77.37	68.46	173.84
	→4)-β-Gal*p*-(1→	104.22	71.68	73.16	76.40	74.36	60.60
	→3)-β-Gal*p*-(1→	103.33	—	80.03	—	—	60.60
	→3,6)-β-Gal*p*-(1→	103.33	—	80.03	—	—	68.33
	→5)-α-Ara*f*-(1→	107.28	80.66	78.89	82.08	66.34	
	→3,5)-α-Ara*f*-(1→	106.95	79.12	83.75	80.72	66.34	
	t-α-Ara*f*-(1→	109.09	81.11	78.97	80.72	60.95	
	→2/2,4)-α-Rha*p*-(1→	98.58	—	—	—	—	16.66/16.39
NGP-2b	→4)-α-Gal*p*A-(1→	98.10	—	—	—	—	174.53
	→4)-β-Gal*p*-(1→	104.22	71.68	73.16	76.40	74.36	60.60
	→5)-α-Ara*f*-(1→	107.28	80.66	78.89	82.09	66.34	
	→3,5)-α-Ara*f*-(1→	106.95	79.12	83.75	80.72	66.34	
	→2/2,4)-α-Rha*p*-(1→	98.16	—	—	—	—	16.66/16.39

#### 3.2.4. Methylation analysis of purified fractions from NGP

Methylation and GC-MS analysis were performed to determine the glycosidic linkages of these RG-I domains. The results of the linkage compositions are shown in table 4. As uronic acid was not reduced prior to methylation, the linkage of GalA was not detected. NGP-1a, NGP-2a and NGP-2b all contained 1,2-Rha*p* and 1,2,4-Rha*p*, which is consistent with the RG-I structure features. 1,2-Rha*p* residues were highly branched at the O-4 position in these fractions, with degree of branching of 86.8%, 62.5% and 54.5%, respectively. The majority of Ara residues present in these fractions were in the form of 1,5-, 1,3,5- and t-Ara*f*, which indicated the abundance of arabinans as side branches of RG-I. High proportion of the 1,4-Gal*p* residues reflected a large number of galactan side chains in RG-I. The glycosyl linkages of 1,3-, 1,6-, and 1,3,6-Gal*p* were indicative of the presence of type II arabinogalactan side chains [25]. These results were consistent with the analysis by NMR spectra.

**Table 4.** The glycosidic linkage type and molar percentage based on methylation and GC-MS analysis.

glycosidic linkage type	NGP-1a	NGP-2a	NGP-2b	1-KGP-P	4-KGP-P
Ara					
t-Ara*f*	17.8	25.2	2.4	1.7	1.2
1,2-Ara*f*	0.4	0.1	—	—	—
1,3-Ara*f*	0.7	0.9	—	—	—
1,5-Ara*f*	13.5	23.3	13.5	2.0	1.4
1,3,5-Ara*f*	15.0	19.7	10.4	2.3	1.1
1,2,3,5-Ara*f*	1.3	0.5	2.9	—	
Gal					
t-Gal*p*	2.8	3.3	1.7	1.3	4.8
1,4-Gal*p*	30.3	12.3	61.3	1.2	2.1
1,3-Gal*p*	5.7	3.0	2.9	1.2	6.4
1,6-Gal*p*	1.9	0.9	0.5	0.6	0.9
1,4,6-Gal*p*	0.3	0.1	0.5	—	—
1,3,6-Gal*p*	7.4	2.6	0.4	1.3	1.6
Rha					
1,2-Rha*p*	0.5	3.0	1.5	—	—
1,2,4-Rha*p*	3.3	5.0	1.8	3.3	1.3
Xyl					
t-Xyl*p*	—	—	—	1.0	3.2
1,4-Xyl*p*	—	—	—	24.4	9.1
Glc					
t-Glc*p*	—	—	—	18.9	3.8
1,2-Glc*p*	—	—	—	7.3	1.4
1,3-Glc*p*	—	—	—	1.5	1.2
1,4-Glc*p*	—	—	—	22.7	19.4
1,6-Glc*p*	—	—	—	3.0	1.4
1,4,6-Glc*p*	—	—	—	5.9	36.6

### 3.3. Purification and structural analysis of 1-KGP and 4-KGP

#### 3.3.1. Enzymatic hydrolysis of 1-KGP and 4-KGP

1-KGP and 4-KGP were both composed of starch-like polysaccharides and hemicellulose. To study the structures of hemicellulose in these fractions, 1-KGP and 4-KGP were hydrolysed by α-amylase. After dialysis, both polymeric fractions (1-KGP-P and 4-KGP-P) and oligomeric fractions (1-KGP-O and 4-KGP-O) were collected. The yields and monosaccharide compositions of these hydrolysis products are listed in table 5. In 1-KGP-O and 4-KGP-O, Glc was the major monosaccharide with a content of 84.8% and 88.7%, respectively, corresponding to oligosaccharides produced by the degradation of starch. The polymeric fractions 1-KGP-P and 4-KGP-P were mainly composed of Glc and Xyl, with minor Gal, Ara, and Man, suggesting they were predominantly composed of hemicellulose such as xyloglucan, xylan or arabinoxylan. The presence of GalA and Rha indicated that small amounts of pectic polysaccharides were also extracted by 1 M and 4 M KOH solution. The FT-IR spectra of 1-KGP-P and 4-KGP-P (figure 2*d,e*) showed typical carbohydrate signals, in which the band at 897 cm**^−^**^1^ was assigned to β-linked sugar residues in hemicellulose [20].

**Table 5.** The yields, molecular weights and monosaccharide compositions of fractions from 1-KGP and 4-KGP.

				monosaccharide composition (w/w %)						
fraction	total sugar (w/w %)	yield^a^ (%)	Mw (kDa)	Glc	Xyl	Gal	Ara	Man	GalA	Rha
1-KGP	68.5	—	—	72.0	9.1	4.3	3.8	6.9	2.0	—
1-KGP-P	75.8	43.2	—	54.5	12.1	9.6	14.6	2.9	3.2	3.2
1-KGP-O	93.5	40.8	—	84.8	6.5	5.2	2.9	—	—	—
4-KGP	68.0	—	—	70.7	12.0	7.5	5.0	2.0	1.7	—
4-KGP-P	73.7	55.1	—	52.9	17.3	11.2	11.7	3.4	1.5	2.0
4-KGP-O	92.6	26.8	—	88.7	3.3	4.6	2.1	1.3	—	—
1-KGP-P1	89.8	25.3	480	5.4	81.5	3.8	3.3	—	3.4	2.5
1-KGP-P2	90.2	63.4	2.7	44.1	19.3	8.0	19.6	—	3.6	5.4
4-KGP-P1	88.7	10.0	446	4.7	37.6	7.9	8.7	—	27.9	13.7
4-KGP-P2	91.1	48.0	123	39.1	26.9	17.7	8.3	—	1.9	—
4-KGP-P3	93.6	23.0	2.6	56.8	12.9	10.6	19.7	—	—	—

^a^Yield in relation to previous fraction.

#### 3.3.2. Purification of 1KGP-P and 4KGP-P by size-exclusion chromatography

**Figure 4.**
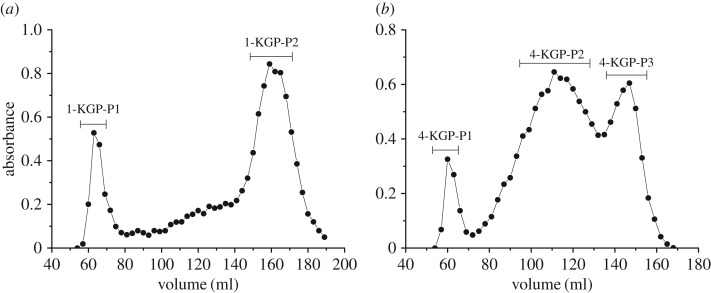
Elution profiles of (*a*) 1-KGP-P and (*b*) 4-KGP-P on Sepharose CL-6B column (filled circles, total sugar).

1-KGP-P and 4-KGP-P were both fractionated by Sepharose CL-6B column. After separation, two fractions, 1-KGP-P1 and 1-KGP-P2, were obtained from 1-KGP-P (figure 4*a*), with molecular weights of 480** **kDa and 2.7** **kDa, respectively. Their monosaccharide compositions are listed in table 5. 1-KGP-P1 was constituted overwhelming by Xyl (81.5%), indicating xylan was predominant in it. 1-KGP-P2 was mainly composed of Glc (44.1%), Xyl (19.3%), Ara (19.6%) and Gal (8.0%). These sugars were attributed to xyloglucan or xylan with Gal or Ara containing side chains. Small amounts of GalA and Rha in 1-KGP-P2 probably arise from co-extracted pectic polysaccharides. 4-KGP-P was separated into three fractions by Sepharose CL-6B, named 4-KGP-P1, 4-KGP-P2 and 4-KGP-P3 (figure 4*b*). Their molecular weights were 446** **kDa, 123** **kDa and 2.6** **kDa, respectively. 4KGP-P1 contained Xyl (37.6%) as the major sugar. Besides, it contained high amounts of GalA (27.9%) and Rha (13.7%), with minor Gal, Ara and Glc. These sugars evidenced the presence of xylan/xyloglucan and pectic polysaccharides which might be associated with each other and co-extracted by 4 M KOH. 4-KGP-P2 and 4-KGP-P3 were both composed of Glc, Xyl, Gal and Ara. Fuc was also detected in 4-KGP-P2. These results indicated both 4-KGP-P2 and 4-KGP-P3 contained different substituted xyloglucan structures.

#### 3.3.3. Enzyme-linked immunosorbent assay detection

**Figure 5.**
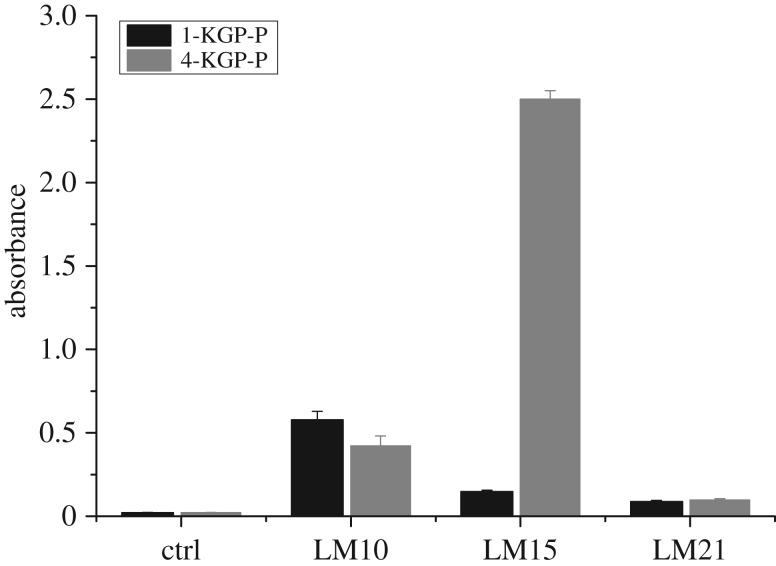
ELISA analysis of the binding of hemicellulose-directed monoclonal antibodies LM10, LM15 and LM 21 to 1KGP-P and 4KGP-P. The values represent the means of triplicate experiments. LM 10 is specific to unsubstituted or low-substituted β-1,4-xylan [26], LM 15 could recognize XXXG structural motif of xyloglucan [27] and LM 21 recognizes hetero-β-1,4-mannan [28].

To detect the structural features of 1-KGP-P and 4-KGP-P, hemicellulose-directed monoclonal antibody (LM 10, LM 15 and LM 21) detection was performed. LM 10 is specific to unsubstituted or low-substituted β-1,4-xylan [26], LM 15 could recognize XXXG structural motif of xyloglucan [27] and LM 21 recognizes hetero-β-1,4-mannan [28]. The binding of 1-KGP-P and 4-KGP-P to LM10 suggested they both contained unsubstituted xylan or low-substituted xylan such as arabinoxylan (figure 5). The binding of LM 15 to 4-KGP-P was stronger than 1-KGP-P, indicating 4-KGP-P contained more xyloglucan epitopes with XXXG structural motifs than 1-KGP-P. Both 1-KGP-P and 4-KGP-P exhibited very weak binding to LM 21, suggesting only a few heteromannans were present in them.

#### 3.3.4. Enzymatic hydrolysis of 1-KGP-P and 4-KGP-P and high performance anion exchange chromatography

**Figure 6.**
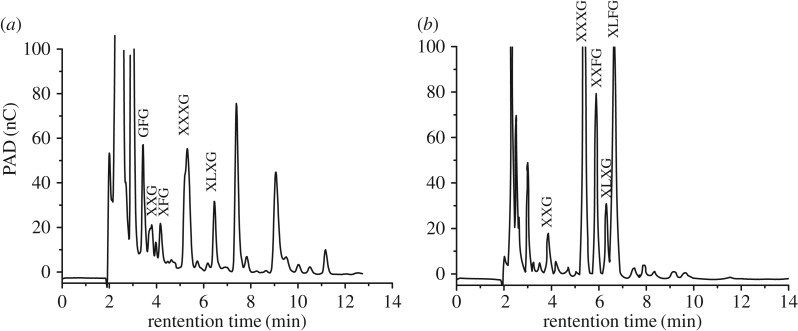
HPAEC analysis of β-glucanase degradation products of (*a*) 1-KGP-P and (*b*) 4-KGP-P.

Xyloglucan is one of the major hemicellulosic polysaccharides. It consists of a backbone of β-1,4-linked-Glc*p* (noted G) of which side chains were attached to O-6 [29]. The main side chains are α-D-Xyl*p*-(1 → (noted X), β-D-Gal*p*-(1 → 2)-α-D-Xyl*p*-(1 → (noted L) and α-L-Fuc*p*-(1 → 2)-β-D-Gal*p*-(1 → 2)-α-D-Xyl*p*-(1 → (noted F). XXXG-, XXGG-, XLFG- and XXFG-type oligomers have been found in endoglucanase-degraded xyloglucan from many plants. 1-KGP-P and 4-KGP-P were degraded by endo-1,4-β-glucanase to further analyse their chemical structures. The released oligosaccharides were detected by HPAEC. Both the HPAEC elution patterns confirmed the presence of oligomers of xyloglucan in 1-KGP-P and 4-KGP-P, which were identified using retention times of known oligosaccharides from apple xyloglucan. As shown in figure 5, the main peaks eluted at 3.4, 3.8, 4.2, 5.3 and 6.5** **min were attributed to GFG, XXG, XFG, XXXG and XLXG oligomers in 1-KGP-P (figure 6*a*), and the elution peaks at 3.9, 5.4, 5.9, 6.3 and 6.6** **min were assigned to XXG, XXXG, XXFG, XLXG and XLFG oligomers in 4-KGP-P, respectively (figure 6*b*). It seemed that 4-KGP-P had more branching than 1-KGP-P. These oligomer structures of xyloglucans were similar to the structures of alkali solution-extracted hemicellulose from subclass *Asteridae* [30] and okra [31].

#### 3.3.5. Methylation analysis of 1-KGP-P and 4-KGP-P

The glycosidic linkages in 1-KGP-P and 4-KGP-P were further analysed by methylation and GC-MS (table 4). The dominant sugar residues in 1-KGP-P and 4-KGP-P were 1,4-Glc*p*, 1,4,6-Glc*p*, t-Glc*p*, 1,4-Xyl*p* and t-Xyl*p*, which were commonly reported in xylan and xyloglucan [29]. 1,4-Glc*p* residues were highly branched at the O-6 position in 4-KGP-P, while less branched at the O-6 position in 1-KGP-P, with degree of branching of 65.4% and 20.6%, respectively. Small quantities of 1,2-Glc*p*, 1,3-Glc*p* and 1,6-Glc*p* were also found in 1-KGP-P and 4-KGP-P. The molar ratio of 1,4-Xyl*p* in 1-KGP-P was higher than in 4-KGP-P. These results suggested xylan was the main component in 1-KGP, while xyloglucan was prevailing in 4-KGP, consistent with ELISA analysis and enzymatic hydrolysis results. Meanwhile, less 1,5-Ara*f*, 1,3,5-Ara*f***,** t-Ara*f*, 1,4-Gal*p*, 1,3-Gal*p*, 1,6-Gal*p*, 1,3,6-Gal*p* and 1,2,4-Rha*p* was also detected in these fractions, which might be due to the co-extraction of pectin with hemicellulose by KOH solution.

### 3.4. Comparison of polysaccharides extracted by different methods from ginseng

Combining the above and previous results, after sequential extraction with hot water, α-amylase, EDTA solution, 50** **mM Na_2_CO_3_ solution, 1 M KOH and 4 M KOH solution, most of the non-cellulosic polysaccharides were obtained from ginseng. These polysaccharides (WGP, WGPE, EGP, NGP, 1-KGP and 4-KGP) account for around 33% in ginseng roots, among which starch-like polysaccharides, pectin and hemicellulose account for approximately 20%, approximately 10% and approximately 3%, respectively. Except for NGP, all other ginseng polysaccharides contained starch. The structural features of all the non-starch polysaccharide fractions are listed in table 6.

**Table 6.** Structural features of non-starch polysaccharide fractions from ginseng. —: not detected; ND: not determined.

	fraction	GalA (%)	GalA + Rha (%)	GalA/ Rha	Glc (%)	Xyl (%)	DM (%)	Mw (kDa)	polysaccharide type
WGP [7]	WGPA-1-RG	1.8	2.0	9.0	3.5	—	—	100	AG
	WGPA-2-RG	5.3	9.3	1.3	2.9	—	—	110	AG
	WGPA-3-RG	20.2	27.5	2.8	3.2	—	ND	310	RG-I
	WGPA-4-RG	38.4	49.8	3.3	4.4	—	ND	330	RG-I
	WGPA-1-HG	62.4	66.0	39.0	7.6	—	30.0	3.5	HG
	WGPA-2-HG	83.6	86.6	27.9	1.9	—	20.0	6.5	HG
	WGPA-3-HG	90.9	92.4	60.6	1.3	—	10.0	16	HG
	WGPA-4-HG	92.1	92.1	184.0	2.0	—	5.0	45	HG
WGPE [12]	WGPE-2a	13.7	22.0	1.7	1.2	—	32.0	430	RG-I
	WGPE-2b	62.2	66.8	13.5	2.9	—	27.0	12	HG
	WGPE-3a	34.9	46.3	3.1	—	—	10.0	420	RG-I
	WGPE-3b	81.7	85.1	24.0	4.1	—	5.0	50	HG
EGP [13]	EGP-2a	32.7	40.7	4.1	3.4	—	1.7	420	HG-RG-I
	EGP-2b	46.5	53.5	6.6	3.9	—	7.6	150	HG-RG-I
	EGP-3a	52.8	61.5	6.1	3.9	—	4.5	430	HG-RG-I
	EGP-3b	64.5	71.9	8.7	6.1	—	3.1	110	HG-RG-I
NGP	NGP-1a	6.3	10.6	1.5	—	—	—	120	RG-I
	NGP-2a	19.1	32.3	1.4	—	—	—	150	RG-I
	NGP-2b	8.7	15.8	1.2	—	—	—	4.9	RG-I
1-KGP	1-KGP-P1	3.4	5.9	1.4	5.4	81.5	—	480	xylan
	1-KGP-P2	3.6	9.0	0.7	44.1	19.3	—	2.7	xylan-xyloglucan
4-KGP	4-KGP-P1	27.9	41.6	2.0	4.7	37.6	—	450	xylan-RG-I
	4-KGP-P2	1.9	1.9	—	39.1	26.9	—	120	xyloglucan
	4-KGP-P3	—	—	—	56.8	12.9	—	2.6	xyloglucan

Structures of different pectic polysaccharides in ginseng were classified according to their contents of GalA and Rha, and the ratios of GalA/Rha. We defined the polysaccharide with (i) the content of GalA > 60% and the ratio of GalA/Rha > 10 as HG; (ii) the content of GalA + Rha > 10% and the ratio of GalA/Rha ≈ 1 to 3 as RG-I; (iii) the content of GalA + Rha > 20% and the ratio of GalA/Rha ≈ 4 to 10 as covalently linked HG and RG-I. Based on these definitions and structural analysis results, WGP was mainly composed of AG (WGPA-1-RG and WGPA-2-RG), RG-I (WGPA-3-RG and WGPA-4-RG) and HG (WGPA-1-HG to WGPA-4-HG) structures. HG fractions were methyl-esterified with degree of methyl-esterification (DM) from 5.0% to 30%. The molecular weights of these pectic polysaccharides were between 3.5** **kDa and 330** **kDa [7]. These free pectins with relatively high DM, which might be located in the middle lamella of the cell walls, could be easily extracted with hot water. WGPE mainly contained RG-I (WGPE-2a and WGPE-3a) and HG (WGPE-2b and WGPE-3b) structures. The DMs of these fractions were between 5.0% and 32.0% [12]. The molecular weights of these fractions were from 12** **kDa to 430** **kDa, higher than those extracted with hot water, suggesting high molecular weights of pectin could be obtained by α-amylase extraction due to the destruction of some starch granules. EDTA-extracted pectin (EGP-2a, EGP-2b, EGP-3a and EGP-3b) contained moderate contents of GalA with lower DM and displayed higher molecular weights (110** **kDa to 430** **kDa). HG and RG-I seemed to be linked together in these fractions [13]. Hence, chelating reagent was thought to release low methyl-esterified pectin which might be bound by Ca^2+^ in the cell walls. Highly branched RG-I-type pectins (NGP-1a, NGP-2a and NGP-2b) were extracted with 50** **mM Na_2_CO_3_ solution. These pectins might be covalently cross-linked to the cell wall and could not be extracted by mild conditions such as hot water or chelator. Na_2_CO_3_ is able to break interpolymeric ester linkages of pectin to other cell wall components [14]. Therefore, we could use Na_2_CO_3_ to obtain these pectins from ginseng which were not extracted before. Unlike above-mentioned pectic polysaccharides in ginseng, the polysaccharides obtained by 1 M and 4 M KOH extraction were mainly hemicellulose. These were in agreement with previous work showing that extraction of dicot cell walls with strong alkali produced predominantly hemicellulose [32]. Xylan was the main component in 1-KGP, while xyloglucan was prevailing in 4-KGP. Xyloglucan-related oligomers XXG, XXXG and XLXG were found in both KOH-extracted hemicellulose fractions. The structural features of these non-starch polysaccharides were consistent with immunocytochemistry analysis of ginseng cell wall in our previous study [18].

## 4. Conclusion

In summary, highly branched RG-I domains were extracted by Na_2_CO_3_ solution, and hemicellulose including xylan and xyloglucan was extracted by KOH solution. As RG-I-type pectin and hemicelluloses have been reported to possess many valuable bioactivities, alkali solution-extracted ginseng polysaccharides might have wide applications in functional food or pharmaceutical industries. These alkali solution-extracted ginseng polysaccharides exhibited great differences from those extracted using other methods. Comparing the polysaccharides obtained by different solvent extractions, we have a comprehensive understanding about total polysaccharides of ginseng.

## Supplementary Material

The primary NMR data for NGP-1a; The primary NMR data for NGP-2a; The primary NMR data for NGP-2b; The primary HPAEC data for β-glucanase degradation products of 1-KGP-P and 4-KGP-P

## Data accessibility

The original data for NMR and HPAEC can be found in the electronic supplementary material.
